# RAWUL: A new ubiquitin-like domain in PRC1 Ring finger proteins that unveils putative plant and worm PRC1 orthologs

**DOI:** 10.1186/1471-2164-9-308

**Published:** 2008-06-27

**Authors:** Luis Sanchez-Pulido, Damien Devos, Zinmay R Sung, Myriam Calonje

**Affiliations:** 1 Centro Nacional de Biotecnología (CNB-CSIC). Cantoblanco, E-28049 Madrid, Spain; 2 EMBL, Meyerhofstrasse 1, 69117 Heidelberg, Germany; 3 Department of Plant and Microbial Biology, University of California, Berkeley, CA 94720, USA; 4 Heidelberg Institute of Plant Sciences, Department of Biodiversity and Plant Systematics, University of Heidelberg, Im Neuenheimer Feld 345, 69120 Heidelberg, Germany

## Abstract

**Background:**

Polycomb group (PcG) proteins are a set of chromatin-modifying proteins that play a key role in epigenetic gene regulation. The PcG proteins form large multiprotein complexes with different activities. The two best-characterized PcG complexes are the PcG repressive complex 1 (PRC1) and 2 (PRC2) that respectively possess histone 2A lysine 119 E3 ubiquitin ligase and histone 3 lysine 27 methyltransferase activities. While PRC2-like complexes are conserved throughout the eukaryotic kingdoms, PRC1-like complexes have only been described in Drosophila and vertebrates. Since both complexes are required for the gene silencing mechanism in Drosophila and vertebrates, how PRC1 function is realized in organisms that apparently lack PRC1 such as plants, is so far unknown. In vertebrates, PRC1 includes three proteins, Ring1B, Ring1A, and Bmi-1 that form an E3 ubiquitin ligase complex. These PRC1 proteins have an N-terminally located Ring finger domain associated to a poorly characterized conserved C-terminal region.

**Results:**

We obtained statistically significant evidences of sequence similarity between the C-terminal region of the PRC1 Ring finger proteins and the ubiquitin (Ubq)-like family proteins, thus defining a new Ubq-like domain, the RAWUL domain. In addition, our analysis revealed the existence of plant and worm proteins that display the conserved combination of a Ring finger domain at the N-terminus and a RAWUL domain at the C-terminus.

**Conclusion:**

Analysis of the conserved domain architecture among PRC1 Ring finger proteins revealed the existence of long sought PRC1 protein orthologs in these organisms, suggesting the functional conservation of PRC1 throughout higher eukaryotes.

## Background

Polycomb group (PcG) proteins are epigenetic gene regulators implicated in important cellular and developmental processes. In animals, PcG proteins were primarily known for their role in maintaining cell identity during the establishment of the body plan [1], but recently they have also been implicated in other important processes like stem cell self-renewal and cancer [2,3]. In plants, the PcG proteins are involved in the repression of flowering during vegetative development [4,5], seed development [6], and in the vernalization response [7]. Hence, PcG proteins play an essential role in the proper development of multicellular organisms.

Biochemical and genetic characterizations of PcG proteins have revealed that they exist in distinct multi-protein complexes [8,9], of which the two best characterized are the PcG repressive complex (PRC)1 and PRC2. The core components of PRC2 are conserved throughout higher eukaryotes. The complex mediates histone H3 methylation at lysine 27 [10-12]. Similar complexes have been found in *Caenorhabditis elegans *[13,14], vertebrates [10-12,15,16] and plants [4,5].

Unlike PRC2, PRC1-like complexes have only been described in Drosophila and vertebrates. Drosophila PRC1 core complex contains Posterior sex combs (Psc), Polyhomeotic (Ph), Drosophila Ring finger protein 1 or Sex combs extra (dRing1/Sce) and Polycomb (Pc) [17,18]. The complex can inhibit in vitro both chromatin remodeling by the human SWItch/Sucrose NonFermentable (hSWI/SNF) complex [19,20] and transcription of a chromatin template by RNA polymerase II [21]. The human PRC1 complex is composed of B lymphoma Mo-MLV insertion region 1 homolog (Bmi-1), PH2, PC3, and the Ring finger proteins (Ring1A/RING1 and Ring1B/RING2), homologs of Drosophila Psc, Ph, Pc, and dRing, respectively. Human Bmi1, Ring1A and Ring1B proteins form an E3 ubiquitin ligase complex that mono-ubiquitinates lysine 119 of nucleosomal histone H2A (H2A-K119) [22,23]. The H2A-K119 monoubiquitination is required for the PcG-mediated in vivo gene silencing, but its role in the mechanism is not yet known [23]. Drosophila PRC1 presumably possesses ubiquitin E3 ligase activity as well, since dRing was found to co-localize with ubiquitinated H2A at the Ultrabithorax (Ubx) promoter [22].

In Drosophila and vertebrates the PcG-mediated gene silencing mechanism requires the action of both PRC2 and PRC1. The apparent lack of homologs of PRC1 components in organisms such as plants or worms and the conflicting information on histone ubiquitination in these organisms led to the speculation of alternative PcG-mediated gene silencing mechanism in which other proteins undertake PRC1 functions [4,24-29]. Ubiquitination at lysine 119 of H2A in human was observed within the consensus peptide sequence PKKT [22]. Arabidopsis genome contains 13 histone H2A genes (*HTA1-13*) [30], among them just HTA10 displays conservation of the PKKT consensus sequence. Although H2A ubiquitination has not been detected in Arabidopsis using western blot analysis so far [31], a recent report showed the presence of ubiquitinated H2A in maize [32]. On the other hand, ubiquitinated H2A has been detected in *C. elegans *[29], indicating that some proteins may be involved in H2A ubiquitination.

The catalytic subunit of the mammalian E3 ubiquitin ligase complex is Ring1B, but Ring1A and Bmi-1 are also required for the in vivo H2A ubiquitination [31]. Bmi-1, Ring1B and Ring1A contain a Ring finger domain located at the N-terminal region. Biochemical analyses showed that the N-terminal Ring finger domain of Ring1B is sufficient for the E3 ligase activity in vitro [22,33,34] and that Bmi-1 displays no detectable ubiquitin ligase activity but it greatly stimulates the E3-ligase activity of Ring1B. It was also described that in vitro Ring1A can replace Ring1B [34]; however, the in vivo function must be distinct, as most of the ubiquitinated H2A is depleted upon lost of Ring1B [31,35]. Structural analysis revealed that Ring1B interacts with Bmi-1 via their Ring fingers [33,34].

The PRC1 Ring finger proteins also share a conserved C-terminal region [36,37] that seems to be implicated in the interaction with Ph and Pc [38-40]. To gain mechanistic insight into the assembly and the enzymatic activity of the PRC1 proteins we performed a detailed computational sequence analysis of the C-terminal region of these proteins. Our findings result in the identification of a new ubiquitin-like domain that unveiled PRC1 Ring finger-like proteins in plants and worm.

## Results and discussion

### Detection of a new ubiquitin-like domain in the PRC1 Ring finger proteins

**Figure 1:**
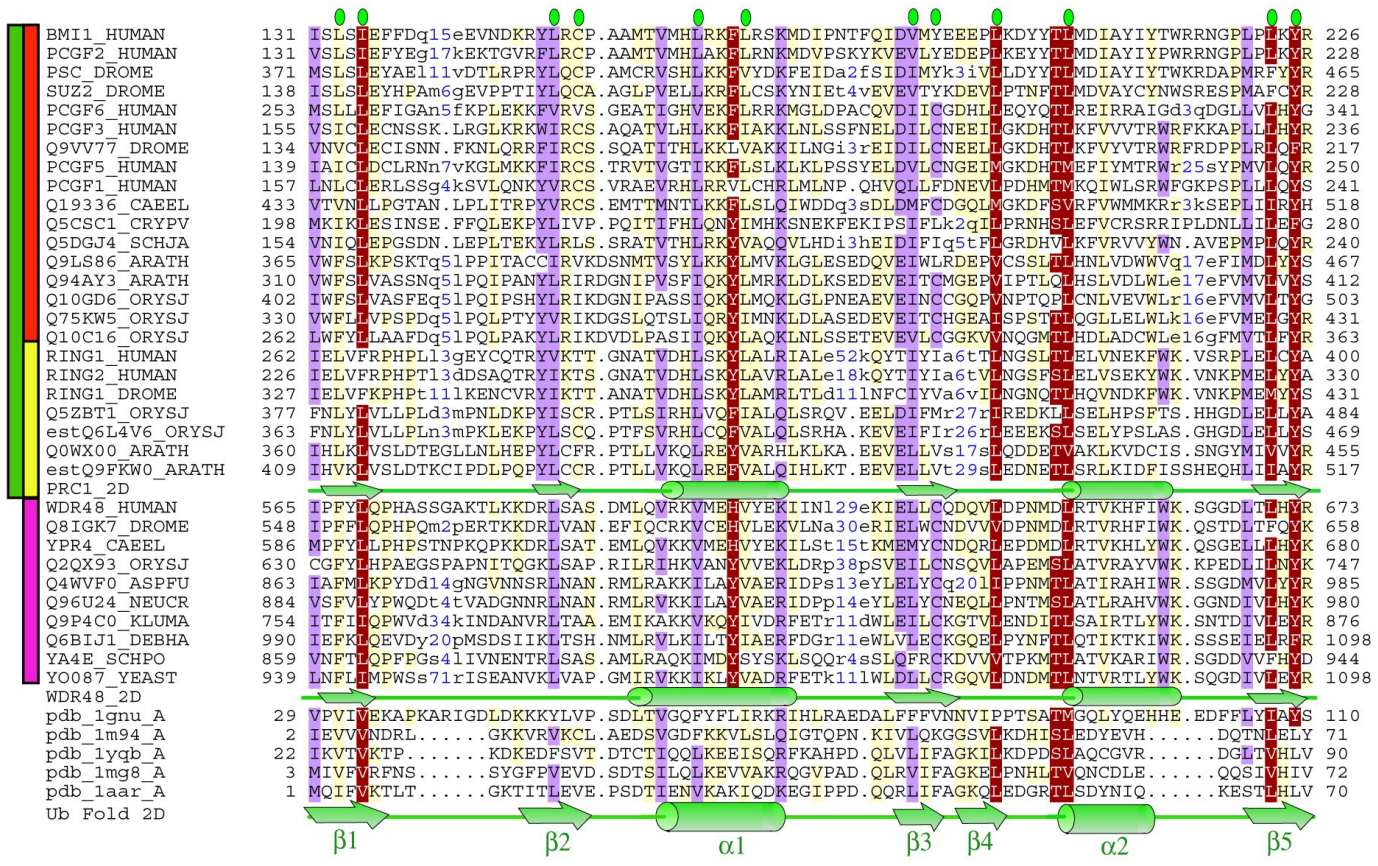
**Representative multiple alignment of the RAWUL domain**. The coloring scheme indicates average BLOSUM62 score (correlated to amino acid conservation) in each alignment column: red (greater than 1.8), violet (between 1.8 and 1) and light yellow (between 1 and 0.2). Residues that are part of the hydrofobic core of the Ubq fold [50] are indicated by green ovals above the alignment. The limits of the domains are indicated by the residue positions on each side. The main families and subfamilies are indicated by coloured bars to the left of the alignment; green (PRC1), violet (WD48-p80), red (Bmi1/Mel18) and yellow (Ring1A/B). Structure based sequence alignment [86] of representative members of UBL superfamily were included: pdb_1gnu: human gamma-aminobutyric acid receptor type A receptor-associated protein (GABARAP) [84]; pdb_1m94: yeast Ubl modifier protein homologous to ubiquitin 1 (Hub1) [87]; pdb_1yqb: human ubiquilin 3 [88]; pdb_1mg8: Ubl domain from mouse Parkin protein [89]; pdb_1aar: bovine Ubiquitin [90]. Independent PHD secondary structure predictions [55] for PRC1 Ring and WDR48-p80 families are shown below the family sequences. Consensus secondary structure of the Ubiquitin superfamily is shown below the alignment [54]. Alpha-helices and beta-strands are indicated by green cylinders and arrows, respectively. The sequences are named with their Uniprot identifications, and also, if necessary, with their common species name: Human, *Homo sapiens*; Drome, *Drosophila melanogaster*; Caeel, *Caenorhabditis elegans*; Arath, *Arabidopsis thaliana*; Orysj, *Oryza sativa; *Crypv, *Cryptosporidium parvum*; Schja, *Schistosoma japonicum*; Aspfu, *Aspergillus fumigatus*; Neucr, *Neurospora crassa*; Kluma, *Kluyveromyces marxianus*; Debha, *Debaryomyces hansenii*; Schpo, *Schizosaccharomyces pombe*; Yeast, *Saccharomyces cerevisiae*. The "est" prefix identifies sequence corrections using consensus sequences manually reconstructed by assembling highly similar expressed sequence tags from identical species (conceptual translations). The numbers in blue within the alignment represent sequence insertions that are not included.

**Figure 2:**
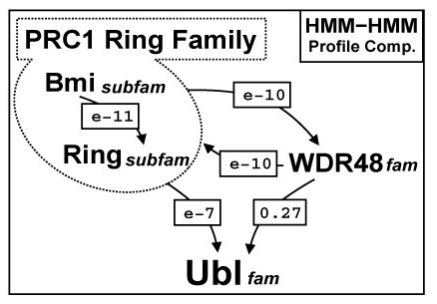
**HMM-HMM profile comparison E-Values between the RAWUL and Ubl domains**. The numbers correspond to HMM-HMM profile comparisons E-values from global profile search results [49] that connect independently each family with the others. The arrows indicate the profile search direction.

**Figure 3:**
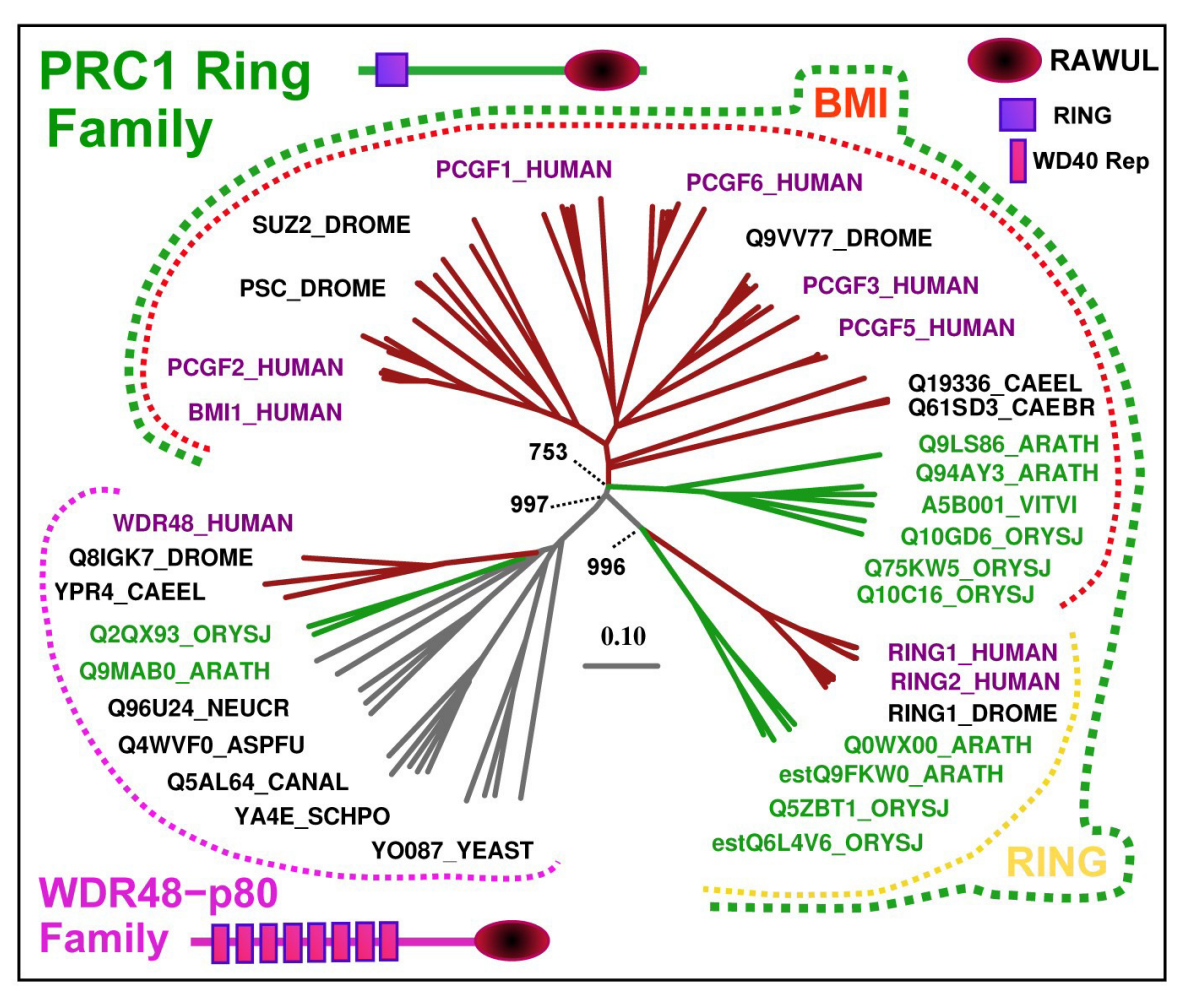
**Dendrogram and Domain Architecture for RAWUL domain containing proteins**. The sequences are named as described in Figure 1. *Vitis vinifera *(Vitvi) sequences were included to construct the dendrogram. The main families (PRC1 Ring and WDR48-p80) and subfamilies (Bmi1/Mel18 and Ring1A/B) are indicated by coloured dotted lines, as in Figure 1. Only a representative set of sequences was labelled. The scale bar shows the average number of amino acid substitutions per site (0.1). The main bootstrap values for PRC1 Ring family classification are indicated. Schematics of domain architecture are represented with the localization of the RAWUL, Ring domains and WD40 repeats according to this analysis, Pfam [91] and REP web servers [92], respectively.

To analyse the conserved C-terminal region of the Ring finger PRC1 proteins, we first performed BLAST searches [41] against different sequence databases located at UniProt [42], NBCI [43], ENSEMBL [44] and JGI [45], starting from the human Bmi-1. Alignments were generated with T-Coffee [46] and checked manually. Additional profile-based sequence searches were performed against the Uniref50 and Uniref 90 protein sequence databases [47] with the defined global hidden Markov models using HMMer [48]. Using extensive profile to sequence (HMMer) and profile to profile comparisons analyses [49] we identified statistically significant E-values of sequence similarity between the C-terminal conserved regions of PRC1 Ring finger proteins and the C-terminal region of the WDR48-p80 protein family (Figure 1 and 2). The WD repeat domain 48 or p80 (WDR48-p80) family are WD40 repeat-containing endosomal proteins found in all eukaryotes including yeast and are present in only one copy per organism (Figure 1 and 3). Except for the previously described interaction between the human WDR48-p80 protein and a tyrosine kinase interacting protein from *Herpesvirus saimiri virus *(Tip), the exact function of the WDR48-p80 family remains unknown but has been suggested to be related to endosome/lysosome traffic [50-52].

The statistical significance of profile to profile comparisons was evaluated in terms of an E-value. Significant E-values connected all these sequences and reciprocal searches produced convergent results (Figure 2). The Bmi1/Mel18 subfamily profile search finds the Ring1A/1B subfamily with a 3.2e-11 E-value and the global profile of the PRC1 Ring family (including both Bmi1/Mel18 and Ring1A/1B subfamilies) detected the WDR48-p80 and the Ubl families with E-values of 3.7e-10 and 4.7e-07, respectively. The RAWUL HMM profile of the WDR48 family have less capability to detect remote protein homologies and localize the ubiquitin family with poor statistical significance (with an E value of 0.21), although RAWUL domain is more conserved inside the WDR48 family than in PRC1 Ring family. Nevertheless, the homology of the WDR48 RAWUL domain with the UBL family is statistically supported, when using as intermediate the RAWUL HMM profile of PRC1 Ring family.

This result defines a new conserved domain present in the PRC1 Ring finger and WDR48 families. In all of those proteins the domain is always observed in a C-terminal location (Figure 3), suggesting that this is somehow important for its function.

Statistically significant sequence similarity offered by profile-profile comparisons linked this domain with ubiquitin superfamily (Figure 1 and 2). Ubiquitin (Ubq) is a highly conserved 76 amino acid residue protein found in all eukaryotic cells and whose sequence is extremely well conserved from protozoa to vertebrates [53]. The overall topology of the Ubq-like fold is β1, β2, α1, β3, β4, α2, β5 (where β indicates beta strand and α alpha helix); however, there may be deviations from this common core [54].

Although those E-values are significant per se, we performed three additional analyses. First, secondary structure predictions were performed independently for the conserved C-terminal regions of PRC1 Ring finger and WDR48-p80 families [55] (Figure 1). These predictions showed good agreement with the Ubq and Ubq-like structures (Figure 1). Second, to investigate whether fold recognition analysis generated consistent results, we submitted the Ubq-like domain of the human Bmi-1 protein (Uniprot-id: BMI1_HUMAN, residues 131 to 226) to an independent fold assignment software (mGenThreader [56]). mGenthreader consistently recognised only Ubq-like proteins as possible templates (8 out of the 10 proposed hits are Ubq-like folds). This consistency in the threading output additionally supports the relationship between Ubq and the conserved PRC1 Ring C-terminal domain. Third, we generated a structural model (see Additional file 1) of the human Bmi-1 putative Ubq-like domain based on alignments including ubiquitin superfamily sequences of known structure (Figure 1). The model was evaluated using statistical mean force potential and found to have a Z-score of -6.5, in the upper range of good model for this length [57], strengthening the assignment of the Ubq fold.

Despite the low sequence identity between this domain and the Ubq (below 20%), the hydrophobic core and key secondary structural elements of the Ubq fold are conserved (Figure 1) [54]. However, the PRC1 Ring finger family displays a higher degree of sequence variability in the C-terminal Ubq-like domain than the WDR48-p80 family. The domain secondary structural elements are rich in charged residues and are separated by loops of variable length (Figure 1 and Additional file 2), which are likely to be the reasons why this domain escaped detection until now. The fact that the domain shows insertions of variable length is not in contradiction with a stable fold [58]; even more, small folds, such as Ubiquitin [54], Immunoglobulin [59] or SBDS [60], share a high degree of structural conservation in a scenario of high sequence divergence and long loop insertions.

The most characteristic features of this domain is the α2 that is a bit larger in its C-terminus compared to classical Ubq-like proteins and contains a conserved pair of Thr/Leu that might have a structural role in capping the alpha helix (Figure 1). Preliminary structural characterization of the C-terminal region of Ring1B showed that this domain folds independently and is a combination of alpha-helix and beta-sheet secondary structures [61], which is in agreement with our analysis.

In summary, considering the profile-profile comparisons E-values (Figure 2), secondary structure predictions (Figure 1), fold assignment detection and the results of the homology model building and evaluation (see Additional file 1), we are confident that this domain is a new member of the Ubq superfamily. Since we found this new Ubq-like domain associated to two different domain architectures, Ring-finger domain (in PRC1 Ring family) and WD40 repeats (in WDR48-p80 family), we named it RAWUL (**R**ing-finger **A**nd **W**D40 associated **U**biquitin-**L**ike) domain.

The ubiquitin related proteins fall into two separate classes [62]. Ubq-like proteins (UBLs; eg, SUMO, NEDD8, etc.) that function as modifiers in a manner analogous to that of ubiquitin and exist either in a free form or attached covalently to other proteins by their C-termini, and the ubiquitin-domain proteins (UDPs) that bear domains related to ubiquitin but are otherwise unrelated in sequence to each other (i.e. parkin, Rad23, DSK2). In contrast to the UBL modifiers, the UDPs are not conjugated to other proteins.

RAWUL domain lacks the characteristic C-terminal diglycine motif required for enzymatic conjugation of the Ubq domain to an acceptor lysine. Neither are conserved the three most common acceptor lysine residues involved in polyubiquitination (Figure 1, represented in ubiquitin (PDB:1aar) as K29, K48, and K63). Hence, it appears unlikely that this domain functions as a traditional UBL modifier. PRC1 Ring finger and WDR48-p80 proteins therefore could be included in the group of UDPs. There are two classes of UDPs as well [63] differentiated by their location and sequence similarity to ubiquitin of their Ubq-like domains.

The Ubq-like domain (UBD) proteins have the Ubq-like domain located at or close to the N-terminus of the protein and defined by a stretch of 45–80 residues with significant sequence homology to ubiquitin, while the Ubiquitin regulatory X (UBX) domain proteins have the Ubq-like domain at the C-terminus consisting of ~80 amino acids that shares common secondary structure organization with ubiquitin despite the lack of significant sequence homology [64,65]. The UBD proteins have been shown to bind to ubiquitin-interacting motifs (UIMs), like parkin protein. Defective parkin protein is responsible for a common familial form of Parkinson's disease. Parkin encodes an E3 ubiquitin ligase that contains two Ring finger domains at its C-terminus and one Ubq-like domain at its N-terminus [65,66]. Binding of the parkin ubiquitin-like domain to the Eps15 UIM is required for parkin-mediated Eps15 ubiquitination [66].

Conversely, only very few UBX domain proteins have been studied in detail and no general function for the UBX domain has yet emerged. Most of the UBX domain proteins identified so far can be grouped into four evolutionary conserved protein families represented by the human Fas-associated factor-1 (FAF1), p47, Y33K, and Rep8 proteins [67]. Recent reports showed that the UBX domains may act as general binding modules for p97 and/or p97 homologs, possibly representing a first common role for UBX domains [68]. RAWUL domain seems to fall into the UBX domain protein family since it is located at the C-terminus, comprise about 80 residues and the conserved hydrophobic core of the Ubq fold. However, as far as we know, UBX domains have not been found together with a Ring finger domain or a WD40 repeat domain.

In the case of the poorly characterized WDR48-p80 family [50-52], the association of WD40 repeats and RAWUL domain could point out to a E3-ligase related function, since WD40 repeats containing proteins are commonly implicated in protein-protein interaction [69] and these repeats have been recently described as key elements in E3 ubiquitin ligase complexes [70].

On the other side, our analysis revealed that the combination of a Ring finger domain at the N-terminus and a RAWUL domain at the C-terminus is a key feature to define the PRC1 Ring finger protein family, since no other protein presents this domain architecture. The Ring finger domains of the Ring (Ring1A and 1B) and Bmi1 proteins are responsible for the E3 ubiquitin ligase activity [33,34], whereas the region corresponding to the RAWUL domain has been shown to mediate protein-protein interaction with Pc and Ph [38-40]. The identification of an Ubq-like domain in this region raise the possibility that these interactions could be establish through a yet uncharacterised UIM present in Pc and Ph. However, due to the structural similarities between the RAWUL domain and ubiquitin, it is also possible that RAWUL could act as an auto-inhibitory domain, by direct competition with the substrate, regulating specificities and/or ubiquitination capabilities of the E3 ligase complex, as previously suggested for Ubq-like domains fused to ubiquitin-specific proteases [71], or as a bridge, linking the proteasome and histone post-translational modifications, as suggested for the Ubq-like domain in the Ubiquitin plant homeodomain Ring finger (UHRF) protein family [72].

### Identification of plant and worm PRC1 Ring finger protein orthologs

**Figure 4:**
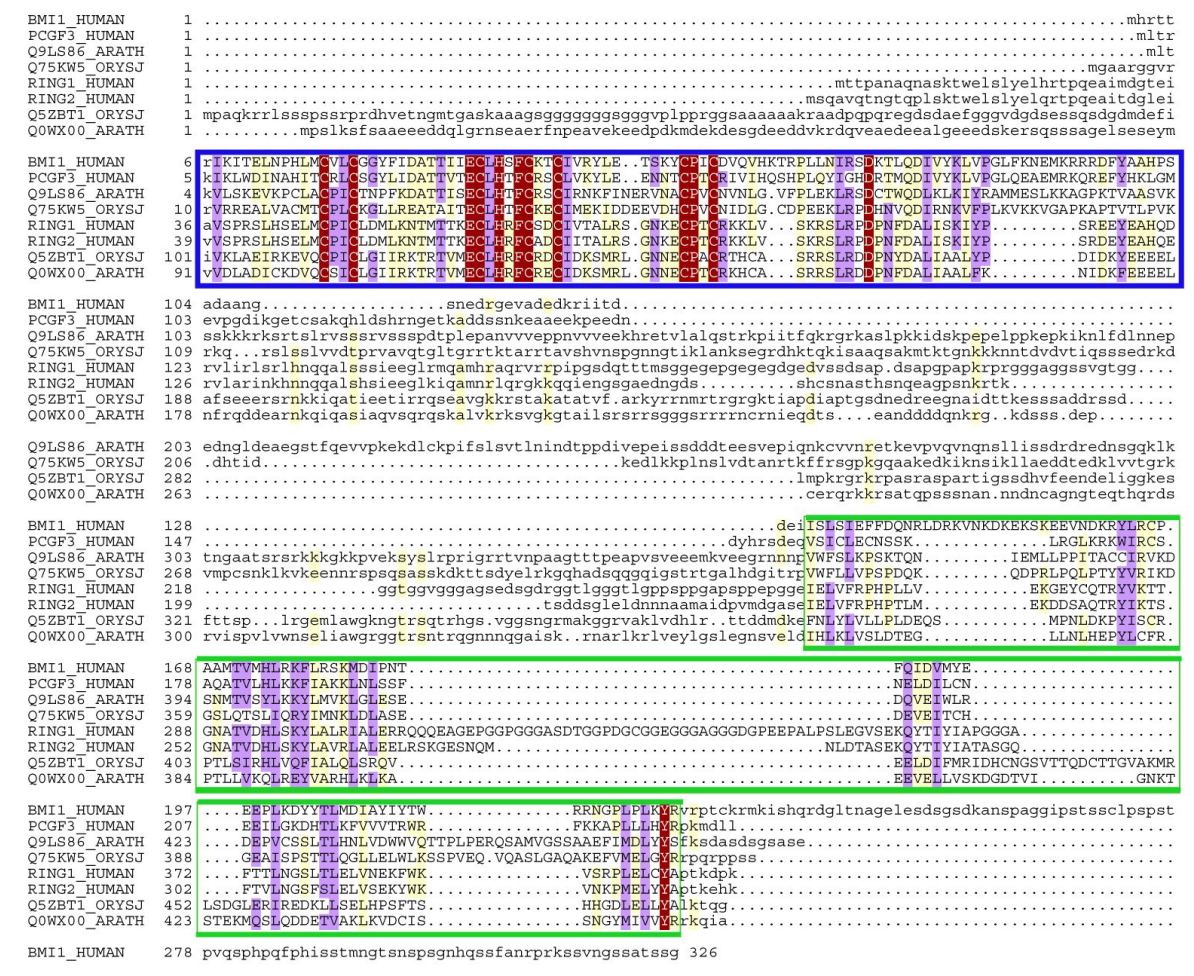
**Representative full sequence alignment of the PRC1 Ring family**. The coloring scheme indicates average BLOSUM62 score (correlated to amino acid conservation) in each alignment column: red (greater than 4), violet (between 4 and 1.5) and light yellow (between 1.5 and 0.5). The sequences are named as described in Figure 1. Lowercase characters represent parts of the sequence that are not evolutionarily conserved. In the PRC1 Ring family the N-terminal Ring domain (blue rectangle) is more conserved than the C-terminal RAWUL domain (green rectangle).

In the course of characterizing the PRC1 Ring finger proteins, we identified a set of proteins in Arabidopsis, *Oryza sativa, Vitis vinifera *and worms that share the PRC1 ring finger domain architecture, an N-terminal Ring finger domain and a RAWUL domain at their C-terminal region (Figure 1, 3 and 4). The preservation of domain architecture among these proteins indicates that these plant and worm proteins are potential orthologs of animal PRC1 Ring finger proteins.

The Ring finger domain is highly abundant in plant proteins, with as many as 469 predicted Ring finger-containing proteins in the Arabidopsis genome [73]. The large number of potential Ring finger proteins in Arabidopsis suggests that target specific ubiquitination plays an important role in protein regulation in plants. However, the combination of the Ring finger domain and the RAWUL domain, which is a specific feature of the PRC1 Ring finger proteins, is found in only a few plant proteins. In Arabidopsis, there are four PRC1 Ring finger genes, At1g03770, At5g44280, At2g30580 and At3g23060 that codify the predicted proteins Q0WX00, estQ9FKW0, Q94AY3 and Q9LS86, respectively. There are full length cDNAs for these four genes with one open reading frame predicted for each (the Arabidopsis information resource (TAIR) database [74]. Online microarray expression data proof that these genes are expressed [74].

Outside of these two domains, the plant proteins have diverged significantly from their putative animal counterparts (Figure 4), but usually PcG protein orthologs do not display sequence homology outside the conserved domains, not only in evolutionary distant organisms but also among paralogs in a given organism [75]. Taking into account the fact that convergent evolution of domain architectures is rare [76], the common domain architecture presented by the plants and animal PRC1 Ring finger proteins (Figure 3) suggest a common evolutionary origin, raising the possibility that these plant proteins could be the PRC1 Ring finger orthologs, in turn implying that the PRC1-mediated histone H2A ubiquitination might also take place in plant.

Plants were thought to have no homologs of PRC1 components. However, two recent reports (25,26) shows that Arabidopsis Like Heterochromatin Protein1 (LHP1, also called Terminal Flower2, TFL2) is functionally similar to Pc, a subunit of PRC1 that recognizes and binds the H3K27 marks created by PRC2. Similarly, PRC1 Ring finger proteins were thought to be missing in plants; however, our analysis revealed the presence of putative orthologs to these proteins. All together these data suggest that PRC1 is also conserved in plants, in agreement with our analysis.

Based on sequence similarity and biochemical data, two groups of PRC1 Ring finger proteins have been defined [18,36], the Bmi1 subfamily that includes vertebrate Bmi1 and Mel18 and Drosophila Psc and Suppressor 2 of zeste (Su(z)2), and the Ring subfamily that includes vertebrate Ring1A, Ring1B and Drosophila dRing. In order to identify potential orthologous relationships between these plant and animal homologous genes we conducted a phylogenetic analysis of this family. Due to the low degree of sequence conservation in the C-terminal domain RAWUL (see Figure 1), we add the N-terminal Ring domain, which is highly conserved between plants and animals (Figure 4), to the phylogenetic analysis of the PRC1 Ring family. The high confidence bootstrap value (997/1000) at the connecting node (Figure 3) indicates that both subfamilies (Bmi1/Mel18 and Ring1A/1B) are clearly present in plants (Figure 3). For instance, Arabidopsis Q0WX00 and estQ9FKW0 proteins are part of the Ring1A/1B subfamily, while Q94AY3 and Q9LS86 proteins are the orthologs of the Bmi1/Mel18 subfamily. The identification of orthologous relationships between the two groups including both plant and animal proteins suggests an early gene duplication event, giving rise to Bmi1/Mel18 and Ring1A/1B subfamilies prior to the plant-animal divergence. The plant and animal proteins therefore could share a common biochemical function.

Similarly, lower metazoans, such as nematodes, are also thought to lack PRC1 homologs. In *C. elegans*, PcG-mediated gene repression has been proposed to require a PRC2-like complex [77] and two novel proteins, Suppressor of Pal-1 (SOP-2) and Sop-2-related protein 1 (SOR-1) [27,28]. Despite the lack of obvious sequence similarity, several conserved properties between PRC1 and the putative SOP-2/SOR-1 complex suggested a conservation of the mechanism [27,28]. However, these proteins are not found in other organisms, including its closest related *C. briggsae *[28], weakening the case for an alternative PRC1-like complex. In addition, ubiquitinated H2A has been detected by western blot analysis in *C. elegans *[31], indicating that some proteins may be involved in H2A ubiquitination. Interestingly, we found one PRC1 Ring finger homolog in *C. elegans *and in *C. briggsae *(Figure 1 and 3). The function of this PRC1 Ring finger homolog is so far unknown. However, the conservation of the protein architecture, an N-terminal Ring finger and a C-terminal RAWUL domain, suggest that the function of this protein could be related to histone ubiquitination.

## Conclusion

We detected statistically significant sequence similarity between the ubiquitin superfamily and the conserved C-terminal regions of PRC1 Ring and WDR48-p80 families, defining a new Ubq-like domain, the RAWUL domain. The identification of an Ubq-like domain in the PRC1 Ring family offers new experimental approaches aimed at elucidating their roles in important cellular processes, such us, stem cell self-renewal and cancer. In addition, characterizing the RAWUL domain allowed us to identify putative PRC1 Ring finger proteins that were thought to be missing in the plants and worms. The possibility that a PRC1-like complex is also involved in PcG-mediated gene silencing mechanism in these organisms is intriguing and opens new avenues in PcG investigation.

## Methods

### Sequence analysis

We first performed BLAST sequence similarity searches [41] against different protein sequence databases: UniProt [42], NBCI [43], ENSEMBL [44] and JGI [45]. For the sequence analysis we related distant protein families via profile searches using global hidden Markov models: HMMer [48] over the UniRef non redundant sequence database [47] and HHpred over pdb70 profile database [49]. The alignments were produced with the T-Coffee and HMMer [46,48] using default parameters, slightly refined manually and viewed with the Belvu program [78]. Dendrogram were calculated with the neighbor-joining method [79] using ClustalW [80] and was edited with Treetool [81]. The stability of different branches with respect to different choices of subsets of residue positions was checked by bootstrapping experiments (1000 replicates) [82]. For profile-profile comparison we used HHpred software (version 1.2.0) downloaded from the authors [49]. Global HMM profiles were done for each family (PRC1 Ring and WDR48-p80) and subfamily (Bmi1/Mel18 and Ring1A/B) using non-redundant alignments of the RAWUL domain (90% of sequence identity) and removing gappy columns (>70%). We include these profiles into the pdb70 HMM-database [49] before profile-profile searches were done. All the profile-profile comparisons were done using default parameters and without known (at Ubl family) or predicted secondary structure information (hhsearch option – ssm 0).

### Protein-structure predictions and modeling

Secondary-structure predictions were performed with Predict Protein server [55]. Fold-recognition analyses were performed with mGenThreader [56]. We generated structural models using Modeler [83] based on pdb structure 1gnu[84]. Models were evaluated using statistical mean force potential [53]. The figure of the model was generated with Pymol program [85].

## Authors' contributions

LSP carried out the database search, sequence analysis and drafted the manuscript. DD carried out the protein structure prediction and modelling, and helped to draft the manuscript. ZRS participated in the coordination and helped to draft the manuscript. MC conceived of the study, and participated in its design and coordination and helped to draft the manuscript. All authors read and approved the final manuscript.

## Supplementary Material

Additional file 1 Homology model of the RAWUL domain from human Bmi1 protein. Click here for file

Additional file 2 Web page with additional information at: Click here for file
