# Supplementary material for: RAWUL: A new ubiquitin-like domain in PRC1 Ring finger proteins that unveils putative plant and worm PRC1 orthologs
Source: BMC Genomics. 2008 Jun 27;9:308. doi: 10.1186/1471-2164-9-308 (PMC2447854; doi:10.1186/1471-2164-9-308)
Supplement: Additional file 1 — Homology model of the RAWUL domain from human Bmi1 protein. [file 1471-2164-9-308-S1.doc]

**
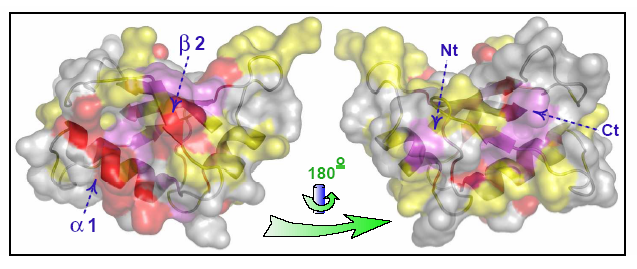
**

**Additional file 1. Homology model of the RAWUL domain from human Bmi1 protein.** The ribbon and surface coloring scheme indicates average BLOSUM62 score (correlated to amino acid conservation) in each residue: red (greater than 3), violet (between 3 and 1.5) and light yellow (between 1.5 and 0.5). For conservation calculation we used a Bmi1/Mel18 subfamily alignment non redundant at 90% of sequence identity. The most conserved secondary structure elements in Bmi1/Mel18 subfamily are label (alpha helix 1 and beta chain 2). N-terminal and C-terminal of the RAWUL domain are indicated. Model was represented with the Pymol program (84).
