# Supplementary material for: RAWUL: A new ubiquitin-like domain in PRC1 Ring finger proteins that unveils putative plant and worm PRC1 orthologs
Source: BMC Genomics. 2008 Jun 27;9:308. doi: 10.1186/1471-2164-9-308 (PMC2447854; doi:10.1186/1471-2164-9-308)
Supplement: Additional file 2 — Web page with additional information at: [file 1471-2164-9-308-S2.htm]

RAWUL


|  |
| --- |
|  |

  
  

### Additional Information about:

### RAWUL: A New Ubiquitin-like Domain in PRC1 Ring finger proteins that unveils putative plant and worm PRC1 orthologs. Luis Sanchez-Pulido, Damien Devos ,Z. Renee Sung and Myriam Calonje Centro Nacional de Biotecnolog�a - CSIC, Spain European Molecular Biology Laboratory, Heidelberg, Germany Department of Plant and Microbial Biology, University of California, USA Heidelberg Institute of Plant Sciences, University of Heidelberg, Germany


---

### - Figure 1 Alignment with Insertions (MUL format) - Update (May - 2008): - List of RAWUL sequences (nonredundant sets) - Arabidopsis on top - - Pdf format - RAWUL Alignment (WDR48 uniref50 set + PRC1 Ring uniref90 set)-(recommended Zoom - 400%) - MUL format - RAWUL Alignment - LAST HHpred Results - RAWUL Domain - RAWUL domain containing sequences(WDR48 uniref50 set + PRC1 Ring uniref90 set) (Fasta Format) - PRC1 Ring Zf Domain - Alignment (pdf format) ---

[CNB]
[Protein Design Group]
[Home Page]


---

  
